# Facility and Regional Variations in Admission and Discharge Patterns Within Step-Up Intermediate Care: A Cross-Sectional Study of Municipal Inpatient Acute Care Services in Norway

**DOI:** 10.1177/11786329241304565

**Published:** 2024-12-04

**Authors:** Fan Yang, Lisa Victoria Burrell, Maren Kristine Raknes Sogstad, Marianne Sundlisæter Skinner

**Affiliations:** Centre for Care Research, Department of Health Sciences in Gjøvik, Norwegian University of Science and Technology (NTNU), Gjøvik, Norway

**Keywords:** Intermediate care, patient transfer, patient admission, patient discharge, health services research

## Abstract

**Background:**

Norwegian Municipal Inpatient Acute Care (MIPAC) services were established as part of the 2012 Coordination Reform. The intention was to prevent unnecessary hospital admissions by redirecting and maintaining less urgent patients at the primary care level, which provides inpatient acute healthcare services closer to patients’ home. However, the role MIPAC plays in the patient trajectory and how trajectories vary across different units and settings is less clear.

**Objective:**

Therefore, this study aimed to (1) describe the general patient transfer trajectories for MIPAC patients and (2) examine facility and regional variations in MIPAC patients’ sources of admission and discharge destinations.

**Design:**

A cross-sectional study using aggregated register data.

**Methods:**

The study involved 36 662 admissions across 185 MIPAC units in 2019. Descriptive statistics were used to describe patient transfer trajectories, and a random-effects multinomial logistic model was applied to assess the association between facility and regional factors and patients’ admission sources and discharge destinations.

**Results:**

The findings revealed distinct admission and discharge patterns based on facility and regional factors. Notably, intermunicipal units with 5 and more municipalities collaborating had higher relative risk ratios (RRR) for discharging to hospital (RRR = 1.50, 95%CI: 1.30-1.72) compared with independent MIPAC units. Large MIPAC units with more than 5 beds had increased relative risk ratios of patients admitted from the hospital than from home (RRR = 4.29, 95%CI: 1.56-11.78). Additionally, regional disparities existed, with units in the Central (RRR = 2.29, 95%CI: 1.56-3.38) and Western Norway health authorities (RRR:1.58, 95%CI: 1.22-2.06) displaying higher nursing home discharge rates than units in the South-Eastern Norway health authority.

**Conclusions and implications:**

This study confirms the Norwegian MIPAC services’ adherence to admission avoidance policies and identifies significant variations in service delivery across regions and facilities. The Norwegian MIPAC model also has potential to inspire other countries in developing admission avoidance services in the primary care setting.

## Introduction

Reducing unnecessary hospitalisation is frequently proposed as a policy measure for curbing growth in health expenditure. Redirecting patients with low acuity to the primary care level has been discussed as a remedy since the 1970s.^1^ In the 2000s, many European countries introduced intermediate care models providing early intervention to serve 2 fundamental purposes: to avoid unnecessary hospital admissions (step-up care), and to facilitate early discharge from hospitals (step-down care).^2-4^ These care models aim to bridge acute, primary and social care, provide a range of services that is more intensive than traditional home or institutional long-term care, but less intensive than acute hospital care.^3,4^

In Norway, the Coordination Reform of 2012 aimed to achieve vertical healthcare integration and seamless patient trajectories by bolstering the role of local authorities – the municipalities.^5^ The Coordination Reform established a mandatory network of governance structures to improve coordination of the decentralised care system, bringing together the health trusts and municipalities to strengthen the coordination between specialist and primary and long-term care services. Municipal Inpatient Acute Care (MIPAC) was introduced as a step-up admission avoidance care model. It provides short-term, round-the-clock accommodation in municipal facilities such as nursing homes, supporting those who require urgent care and would otherwise be admitted to hospital.^6^ The aim of the service is to provide timely inpatient acute care closer to patients’ homes and avoid unnecessary hospital admissions.^7^ Patients must be assessed and referred to the service by a primary care practitioner.^8^ Most referrals are made by general practitioners (GPs) in either daytime or out-of-hours services,^9^ but it is up to the nurses (and if present, the physician) at the individual MIPAC units to decide on admissions, which often depend on capacity and fit with the unit’s inclusion and exclusion criteria.^10^ Medical staff decide when the patient is ready for discharge. In cases where the patient is in need of post-discharge care, healthcare staff and administrative personnel in the municipality are involved in needs assessment and allocation of long-term care services – institutional or home care.^10,11^ Generally, the MIPAC service’s target patients are those frequently admitted to hospitals, but who do not require specialist care. This typically includes elderly individuals and patients with worsening chronic conditions, as well as those with acute or established disorders.^8^ In 2019, patients over 66 years old accounted for 70% of the total MIPAC patients.^9^ In 2016, the service became statutory; all municipalities are required to provide MIPAC services to their residents.^8^

According to the original policy, the ideal trajectory for a MIPAC patient is admission from the community and discharge back to the community, avoiding transfer to the hospital altogether. The Norwegian Health Authorities’ guidelines stated that suitable candidates referred to MIPAC by primary care physicians were patients who were stable, with or without a definite diagnosis, expected to be fully treated within 3 days of monitoring and treatment, and then discharged to the community.^8^ Current knowledge about why community transfer rates vary across municipal and organisational contexts remains limited. Assessing geographical and other variations in where patients come from and are discharged to is necessary to understand the role of the MIPAC service in the healthcare system – whether it functions as an admission avoidance model (step-up only) or a mixed model of intermediate care (including step-up and step-down care). This enables enhanced understanding of the care chain, factors that impact on the access to care and identifying desirable and undesirable variations in admission and discharge patterns.^12-14^ Four studies have reported transfer rates from MIPAC services to hospitals ranging from 7.5% to 23.6%,^15-18^ and this rate may suggest a risk of delay in receiving appropriate treatment.^17^ However, existing research is fragmented, with studies so far only focusing on individual MIPAC units.^15-17^ These units varied significantly in size, from as few as 2 beds (with a 7.5% hospital transfer rate)^16,17^ to as many as 34 beds (23.6% hospital transfer rate).^15^ These findings highlight the need for further investigation of variations in patient trajectories across MIPAC units in Norway.

Recognising variations in patient trajectories, including where they are admitted and discharged to, is also essential for enhancing collaboration and patient management across diverse care providers within the healthcare system.^19-21^ Previous research has identified that patients, hospital, and regional characteristics are associated with patient admission sources and discharge destinations.^22-25^ Most of this research has focused on emergency and hospital care, while few studies have focused on intermediate care, particularly for step-up intermediate care facilities, such as MIPAC.

There is considerable variation in how the MIPAC units are organised.^26,27^ Municipalities can either manage the unit independently or collaborate with neighbouring municipalities to establish an intermunicipal MIPAC unit located in one host municipality.^26,27^ The most common location for the service is nursing homes, followed by GP out-of-hours clinics, institutions specialising in short-term intermediate care and so-called local (or district) medical centres.^26,27^ There is also considerable regional geographical variation in Norway. The northern parts of the country are sparsely populated and have a geography that challenges transport and communication, while the southern parts are more densely populated.^28^ Norway’s 4 Regional Health Authorities (RHAs) are responsible for specialised care, while the local authorities – the municipalities – organise primary and long-term care. The 4 RHAs cover the South-Eastern, Western, Central and Northern regions of Norway, each anchored by 1 or 2 metropolitan centres. The South-Eastern Norway RHA is the largest; it serves a population of approximately 3.1 million and has the highest population density.^29^ In contrast, the Northern Norway RHA serves a population of around 480 000 and has the lowest population density.^29^ The number of hospitals is positively correlated with population density, but the number of beds per 100 000 inhabitants in South-Eastern RHA is lower than in the North.^30^

We hypothesised that MIPAC facilities’ characteristics and regional variations play a vital role in how the MIPAC service is used. Our focus on using admission sources and discharge destinations to describe the patient trajectory was driven by their critical role in understanding care transitions within the healthcare system. Admission sources provide insights into where patients come from, which is essential for understanding referrals and admissions to the service and what role the service plays in the care system. Discharge destinations, on the other hand, are closely aligned with outcomes of patient care. Together, these variables offer a view of how patients move through the MIPAC units and insights into the service’s function in the care system. Our analysis aims to provide new and generalisable knowledge by describing the general patient trajectories using national publicly available data. The study offers a fresh perspective by investigating associations between facility and regional factors and patients’ trajectories, providing insights for future policy and research.

## Methods

This cross-sectional study used register data aggregated at the MIPAC level to investigate admissions from and discharges to home, nursing homes, other municipal facilities and hospitals.

### Data

Information on the MIPAC facility characteristics and trajectories of MIPAC units in 2019 was obtained from the Norwegian Directorate of Health (Helsedirektoratet) MIPAC administrative data. This data was collected by means of survey from all MIPAC units by the Directorate of Health.^31^ It contains patient admission sources and discharge destinations in each MIPAC unit, as well as the number of MIPAC beds and information on inter-municipal collaboration.

Information on municipalities was obtained from Statistics Norway’s KOSTRA database,^32^ encompassing the population size, proportion of residents aged over 80 years and Regional Health Authorities to which each municipality belonged. To ensure that our dataset was aligned with the MIPAC unit catchment regions, we aggregated the data from the municipality level to the MIPAC level using the 2019 municipality number.

In the MIPAC administrative data file, each MIPAC unit was assigned a unique MIPAC identification number that linked it to its respective municipalities, which enabled merging with KOSTRA data. The file reported on 422 municipalities (including 4 without a MIPAC identification number), and a total of 215 MIPAC units. In the survey, MIPAC admission and discharge information were reported separately, and there were some missing data. For example, some municipalities reported data on admission sources, but not discharge destinations, and vice versa. According to the MIPAC status report, the most common cause of missing data was that certain information was not available to all municipalities, possibly due to insufficient data registration routines and/or difficulties in extracting statistics from their systems.^9^ In 2019, 185 MIPAC units (corresponding to 363 municipalities) reported discharge information (accounting for 86% of the 215 MIPAC units), representing 36 662 discharges, and 176 units reported admission sources, representing 35 158 admissions. To maximise the use of available data, we included the 185 MIPAC units that reported discharge data, and the 176 units that reported admission data and ran separate analyses for admission and discharge patterns.

We also examined the 30 MIPAC units missing discharge information and the 39 units missing admission information. A chi-square test revealed no significant differences between the excluded MIPAC units and those included in terms of RHA affiliation or the number of residents in their catchment areas.

### Study variables

Our focus was on 2 primary outcomes: (1) admission sources, defined as the origin of patient admissions and (2) discharge destinations, representing the location patients moved to after the MIPAC stay. Both admission sources and discharge destinations included 4 settings: home (with or without municipal home care services), nursing home, other municipal facilities and hospital. To emphasise the distinction between primary and specialist healthcare systems, we further conceptualised the first 3 settings as ‘community’ settings and the latter as hospital. Community admission and community discharge thus refer to MIPAC patients who are admitted from or discharged to the following 3 community care settings: home (with or without municipal home care services), nursing home, and other municipal facilities.

To analyse the variation at the MIPAC level, we examined *intermunicipal collaboration, MIPAC unit bed counts, location, RHA affiliation, catchment population, share of residents over 80* *years old in the MIPAC unit’s catchment area (shortened to* ‘*share of residents over 80 years old*’*);* and *travel distance by car between the MIPAC’s host municipality and the nearest emergency department (ED) in kilometres.*

The thresholds for *intermunicipal collaboration* (2-5 and >5 municipalities collaborating) and *MIPAC unit bed counts* (≤2, 2<beds≤5 and >5 beds) were selected for easier interpretation and comparison. The *intermunicipal collaboration* indicated whether MIPAC unit organise the service independently or collaboratively. *Location* provided information on where the municipalities located their MIPAC units and encompassed nursing home, GP out-of-hours clinic, municipal intermediate care institutions, other facilities and multiple locations. The *MIPAC unit’s catchment population* and *the share of residents over 80* *years old* will reflect the overall demand for health care services. An early study of MIPAC found that an increase in the share of the population with higher age was associated with increased hospital admission rates.^33^ Thus, we include the share of residents over 80 years old in the MIPAC’s catchment area to assess whether the overall demand for healthcare services is associated with MIPAC admission and discharge patterns. Another study found that long distance between the MIPAC unit and the nearest hospital was a major barrier to intermunicipal cooperation on MIPAC.^34^ Thus, in our study, we included the distance between the MIPAC host municipality and the nearest ED as a proxy for the distance to the hospital.

To make the results and the interpretation more accessible, the continuous variable representing the share of residents over 80 years old was modelled with each one-percentage-point increase. Similarly, the travel distance from the MIPAC host municipality to the nearest ED was modelled with each 10 km increase.

### Statistical analysis

We reported descriptive statistics for the included variables at the MIPAC and discharge (patient) levels, computing proportions for categorical variables and median with interquartile range for continuous variables. Additionally, rank scatterplots were generated to visualise the variation in the rates of community admission and discharge across the 4 regions. We fitted 2 separate random-effects multinomial logit models to quantify the associations between the included independent variables and patients’ admission sources and discharge destinations, respectively. This approach is suitable given the different types of admission sources and discharge destinations, with patients nested within each MIPAC unit. Random-effects multinomial logit models are used to fit random-effects for a categorical dependent variable with unordered outcomes.^35^ Unobservable heterogeneity at the MIPAC level was accounted for by a random effect^35^ and we did not impose an independent covariance structure for the random effect to achieve a more accurate estimate.^36^ The findings are reported as relative risk ratios (RRRs) with a 95% confidence interval (CI). All analyses were performed using STATA 18.0^36^ and R studio.^37^

### Ethical approval

This study was conducted in accordance with the ethical principles of the World Medical Association Declaration of Helsinki.^38^ This study used only aggregated data from the Norwegian Ministry of Health and Statistics and did not require ethical approval. All the data are publicly available and can be accessed online.

## Results

Table 1 shows that 19 562 of 36 662 discharges (53%) were treated across 127 independent MIPAC units, while the remaining discharges were managed in 58 intermunicipal MIPAC units. Most MIPAC units were relatively small, hosting fewer than 2 beds (62%); however, over half of the discharges (56%) were treated in larger MIPAC units equipped with more than 5 beds. Although more than half of the MIPAC units (57%) were located in nursing homes, the distribution of discharges was uniform across the 5 locations. In total, 25 MIPAC units (14%) had catchment areas with more than 50 000 residents, accounting for 54% of the total discharge. The South-Eastern Norway Regional Health Authority (RHA) had 34% of MIPAC units and treated half of the discharges. Residents aged over 80 years accounted for 5.2% of the total population in the MIPAC unit catchment area. The median travel distance between the MIPAC’s host municipality and the nearest ED was 42 km, with interquartile range from 16.2 to 86.8 km.

**Table 1.** Overview of organisational characteristics for MIPAC units and discharges.

Study variables	MIPAC units (N = 185)	%	Discharges (n = 36 662)	%
*Intermunicipal collaboration*				
Independent MIPAC units	127	69	19 562	53
Intermunicipal MIPAC units	58	31	17 100	46
(1) 2-5 municipalities collaborate	44	24	10 416	28
(2)>5 municipalities collaborate	14	8	6684	18
*MIPAC unit bed counts*				
⩽2 beds	115	62	7075	19
2<beds≤5	44	24	9130	25
More than 5 beds	26	14	20 457	56
*Location* ^a^				
Nursing home	106	57	7931	22
GP out-of-hours clinic	17	9	9048	25
Municipal intermediate care institutions	26	14	7594	21
Other municipal facility	11	6	3969	11
Multiple locations	25	14	8120	22
*MIPAC unit’s catchment population*				
<5000	65	35	3789	11
5000-9999	32	17	2653	18
10 000-19 999	31	17	4116	7
20 000-49 999	32	17	6437	10
⩾50 000	25	14	19 667	54
*Regional Health Authorities (RHA)*				
South-Eastern Norway RHA	63	34	18 571	51
Western Norway RHA	35	19	7325	20
Central Norway RHA	36	19	4109	11
Northern Norway RHA	52	28	6657	18
*Share of residents aged over 80 (%)*				
25th percentile	4.4			
Median	5.2			
75th percentile	6.0			
*Travel distance between MIPAC host municipality and the nearest ED (km)*				
25th percentile	16.2			
Median	42			
75th percentile	86.8			

a Nursing home: ‘Sykehjem’ in Norwegian; GP out-of-hours clinic: ‘Legevakt’ in Norwegian, which provides GP services after hours; Municipal intermediate care institutions: LMS/DMS/helsehus in Norwegian, so-called Local medical centre/District medical centre/‘Health houses’, which mainly provide short-term stays and, in some places, decentralised specialist health services.

**Figure 1.**
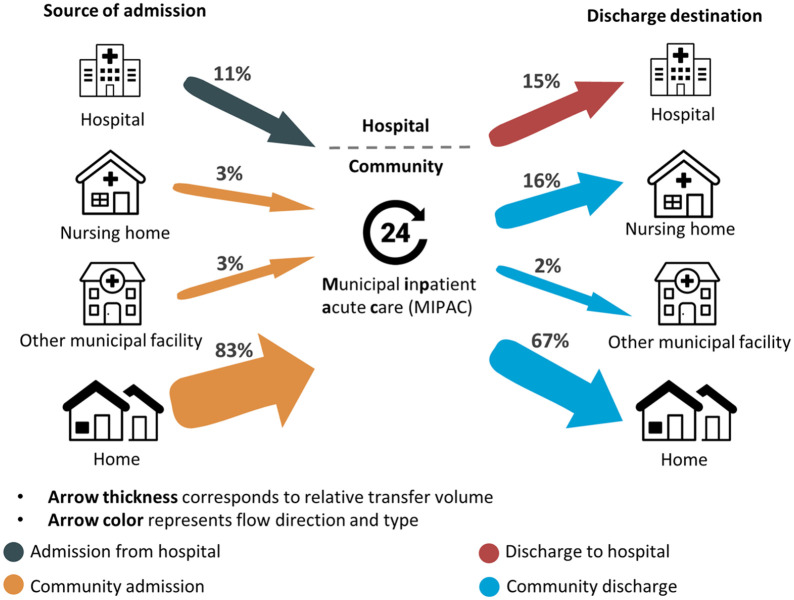
Patient transfer sources and destinations for the MIPAC service.

Figure 1 shows the transfer trajectories of patients and the percentages admitted from and discharged to the different settings. Overall, home was the main admission source and discharge destination, although there was a reduction in the proportion of patients discharged to home compared to those initially admitted from home. Conversely, the proportion of patients discharged to nursing homes and hospitals was higher than that of patients initially admitted from nursing homes (3% vs 16%) and hospitals (11% vs 15%).

**Figure 2.**
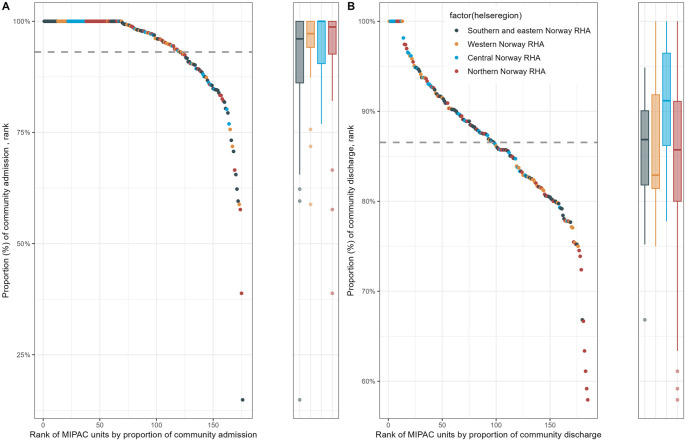
Rate of MIPAC (A) community admission (n = 176) and (B) community discharge (n = 185), by Regional Health Authority (RHA).

Figure 2 presents the analyses of regional variations in community admission and discharge. Among the 176 MIPAC units that reported the source of admission in 2019, community admission rates varied from 14.9% to 100%. The average rate of community admission was 93.1% (dashed line in Figure 2A), while 120 (68.2%) MIPAC units had a higher rate than the average, including 69 MIPAC units with a 100% community admission rate. The right panel of Figure 2 shows that the community discharge rates for the 185 MIPAC units ranged from 57.9% to 100%, with an average of 86.5% (dashed line in Figure 2B). A total of 73 (39.5%) MIPAC units displayed significantly lower community discharge rates than the average, while 84 (45.4%) MIPAC units had significantly higher community discharge rates than the average (*P* < .05).

Although Figure 2 shows that there were some variations among the 4 RHA, the median community admission rate in each region was higher than the national average (93.1%). Variation was also found in the community discharge rates among the 4 RHA, with the highest median community discharge rate found in the Central Norway RHA and the lowest median rate found in the Western Norway RHA, which was lower than the national average (86.5%).

Table 2 presents the relative risk ratios (RRRs) of the random effects multinomial logit models for admission and discharge destinations. The MIPAC unit’s catchment population variable was excluded from the model because it had a high correlation with the MIPAC unit bed count and travel distance between MIPAC host municipality and the nearest ED, violating the assumption of multicollinearity (see Supplemental File 1). The model fits better than the pooled multinomial logit model (likelihood ratio test *P* < 0.05; see Supplemental File 2). The random effects are correlated in the admission model (likelihood ratio test *P*  < 0.05) and partially correlated in the discharge model (likelihood ratio test *P*  > 0.05, while correlation between hospitals and other municipal facilities is significant at the 10% level).

**Table 2.** Relative risk ratios (RRRs) of random effect multinomial logit models with unstructured covariance.

	Admission RRR (95%CI) vs home			Discharge RRR (95%CI) vs home		
Study variables	Nursing home	Hospital	Other municipal facility	Nursing home	Hospital	Other municipal facility
Intermunicipal collaboration (ref: independent MIPAC units)						
2-4 Municipalities collaborate	1.11 (0.53,2.33)	1.11 (0.51,2.41)	0.09 (0.02,0.34)*	0.72 (0.55,0.94)*	1.37 (1.18,1.59)*	0.36 (0.18,0.69)*
⩾5 Municipalities collaborate	0.31 (0.11,0.90)*	0.95 (0.49,1.84)	0.19 (0.06,0.65)*	1.93 (1.54,2.42)*	1.50 (1.30,1.72)*	0.26 (0.12,0.53)*
MIPAC unit bed counts (ref: ⩽2 beds)						
2<beds≤5	0.79 (0.33,1.87)	2.16 (0.83,5.63)	1.66 (0.33,8.40)	0.52 (0.32,0.86)*	1.07 (0.86,1.33)	1.26 (0.51,3.11)
More than 5 beds	0.39 (0.15,1.00)	4.29 (1.56,11.78)*	7.88 (1.36,45.88)*	0.44 (0.27,0.72)*	0.97 (0.77,1.22)	0.88 (0.35,2.21)
Location (ref: Nursing home)						
GP out-of-hours clinic	0.49 (0.19,1.26)	1.20 (0.46,3.12)	2.87 (0.55,15.00)	1.66 (1.03,2.68)*	1.13 (0.90,1.41)	1.88 (0.78,4.49)
Municipal intermediate care institutions	2.09 (0.77,5.73)	0.82 (0.26,2.59)	3.25 (0.50,21.21)	1.03 (0.66,1.61)	0.80 (0.64,1.00)	2.19 (0.88,5.43)
Other facility	2.15 (0.81,5.69)	7.05 (2.67,18.60)*	8.82 (1.47,53.09)*	0.44 (0.28,0.70)*	1.13 (0.91,1.41)	1.78 (0.72,4.40)
Multiple locations	1.43 (0.57,3.59)	0.97 (0.35,2.71)	1.19 (0.25,5.68)	0.35 (0.23,0.55)*	1.02 (0.83,1.26)	0.99 (0.43,2.29)
RHA (ref: South-Eastern Norway RHA)						
Western Norway RHA	1.48 (0.72,3.03)	0.66 (0.34,1.31)	0.14 (0.04,0.49)*	1.58 (1.22,2.06)*	1.32 (1.14,1.52)*	0.72 (0.38,1.39)
Central Norway RHA	1.82 (0.78,4.28)	0.64 (0.24,1.70)	0.10 (0.02,0.60)*	2.29 (1.56,3.38)*	0.86 (0.70,1.07)	2.20 (1.01,4.79)*
Northern Norway RHA	1.08 (0.44,2.68)	0.56 (0.21,1.50)	0.15 (0.03,0.83)*	1.25 (0.76,2.07)	1.10 (0.86,1.40)	1.49 (0.59,3.75)
Share of residents aged over 80	1.13 (0.88,1.47)	0.78 (0.59,1.04)	1.01 (0.63,1.62)	1.09 (0.95,1.25)	1.01 (0.94,1.09)	0.94 (0.71,1.24)
Travel distance between MIPAC’s municipality and the nearest ED (10 km)	1.01 (0.95,1.07)	1.06 (1.00,1.13)	1.15 (1.04,1.28)*	0.95 (0.92,0.98)*	1.02 (1.01,1.04)*	1.02 (0.96,1.08)
Constant	0.01 (0.00,0.04)*	0.05 (0.01,0.26)*	0.14 (0.01,0.03)*	0.26 (0.11,0.59)*	0.15 (0.10,0.22)*	0.02 (0.00,0.08)*

* *P* < .05.

The findings showed that both admission and discharge destinations varied across intermunicipal collaborations. There was a significantly lower RRR of being admitted from (RRR: 0.31, 95%CI [0.11,0.90]) and a higher RRR of being discharged to (RRR: 1.93, 95%CI [1.54,2.42]) nursing homes than to home for intermunicipal MIPAC units with 5 or more municipalities collaborating, compared with independent MIPAC units. Patients admitted to intermunicipal MIPAC units (both 2-4 and more than 5 municipalities collaborating) had a significantly higher RRR for discharge to hospital versus home than those admitted to independent MIPAC units.

In comparison to smaller MIPAC units (⩽2 beds), larger MIPAC units had a significantly lower RRR of discharging patients to nursing home than home. Larger MIPAC units with more than 5 beds had a significantly higher RRR of admitting patients from hospital than home compared to smaller MIPAC units (⩽2 beds; RRR:4.29, 95%CI [1.56,11.78]).

Compared with MIPAC units located in nursing homes, those located in other facilities had a significantly increased RRR for patients admitted from hospitals or municipal facilities other than home. However, there was no significant variation in the discharge destination between MIPAC units located in nursing homes and those located in other facilities. Patients admitted to MIPAC units located in GP out-of-hour clinics had a significantly higher RRR for discharge to nursing homes than those treated in nursing home-based MIPAC units (RRR: 1.66, 95%CI [1.03,2.68]).

The health region to which MIPACs belonged was significantly associated with the discharge destinations. Compared with MIPAC units in the South-Eastern RHA, MIPAC units in the Western (RRR: 1.58, 95%CI [1.22,2.06]) and Central RHAs (RRR: 2.29, 95%CI [1.56,3.38]) had significantly higher RRRs for referring patients to nursing home than home. MIPAC units in the Western RHA (RRR: 1.32, 95%CI [1.14,1.52]) had significantly higher RRR to refer patients upward to the hospital than to discharge them to home.

We did not find any significant associations with the proportion of residents aged >80 years.

As the travel distance between MIPAC host municipality and the nearest ED increased, the relative risk to refer patients to nursing home than home decreased, while the relative risk to refer patients to hospital than home after discharge increased.

## Discussion

Avoiding unnecessary hospital admissions by ensuring timely and accessible treatment and care close to home was the primary purpose for establishing Norwegian MIPAC services. This national-level observational study reveals that community admission and discharge rates vary across MIPAC units and health regions. The study also identifies MIPAC facility and regional factors associated with variations in where MIPAC patients are admitted from and discharged to.

One of the key findings from our study was that, in line with the policy’s intention, most patients were admitted and discharged to the community (home or nursing home; see Figure 1). However, it is noteworthy that several MIPAC units seem to admit a high percentage of patients from the hospital, and some discharge a high percentage of patients to the hospital after a MIPAC stay (see Figure 2). From our data, we are unable to infer the extent to which step-down admissions from the hospital are referrals from the emergency department aimed at preventing hospital admission or if they are transfers for additional care following hospital discharge. Fifteen percent of patients in our study were upwardly transferred to the hospital, suggesting that the medical and nursing staff at the services ensure that patients are transferred when necessary to receive hospital care.

To explain some of the variation we found in community admission and discharge rates across MIPAC services, we explored the associations between facilities and regional characteristics and where MIPAC patients were admitted from and discharged to (see Table 2). We found that intermunicipal MIPAC units had a higher relative risk of discharging patients to hospital than to home, compared to independent MIPAC units. Intermunicipal cooperation often enables small and sparsely populated municipalities to pool resources, consolidating expertise, equipment and funding to address capacity challenges and offer more robust services.^39,40^ A previous study on intermunicipal cooperation in GP out-of-hour clinics identified a pronounced negative effect on workforce quality (measured by the unweighted sum of personnel on duty) when collaboration expanded to include 2 municipalities; however, the effect became positive as the number of collaborating municipalities increased.^41^ Another aspect of intermunicipal cooperation is that it often increases the complexity of coordination between different services and actors, which can pose challenges to efficient healthcare delivery.^42^ For instance, increased travel distances in intermunicipal MIPAC units may produce negative health outcomes, particularly for older adult patients, and potentially result in more upward hospital transfers.

Another important finding of our study was that larger MIPAC units (with more than 5 beds) had an increased relative risk of accepting patients from the hospital rather than from home. This indicate that larger MIPAC units are more likely to be used for step-down care. Larger MIPAC units are likely to have higher nursing competence^43^ and more efficient capacity planning, enabling them to accommodate a more complex patient mix. This enhanced capability might enable them to accept patients discharged from a hospital ward or those who have been ‘turned around’ in the emergency department.

Our study suggests that MIPAC services in the Western region are more inclined to transfer patients to hospitals than to home, compared to the South-Eastern health region. This indicates a regional variation in MIPAC service utilisation. The fact that MIPAC patients in the West are transferred to the hospital to a larger extent may indicate regional disparities in how GPs use MIPAC services. The Western region in Norway is characterised by long distances to urban areas, the nearest hospital and challenging travel conditions (with mountainous areas, fjords, and islands that rely on ferry transport). One explanation could be that MIPAC personnel in the Western RHA have a lower threshold for transferring patients to the hospital if they see signs of deterioration because they know that the journey to the hospital takes a long time. Few studies have investigated regional variations in MIPAC service utilisation. One study found that the Central and Northern health regions exhibited lower nursing competence in MIPAC compared to the South-Eastern and Western health regions.^43^ Furthermore, another study showed that the hospital regional effect accounted for about half of the difference in average health care utilisation.^44^

A 10 km increase in travel distance between the MIPAC host municipality and the nearest ED was associated with a slightly higher relative risk of discharging patients to the hospital rather than home, and a slightly lower relative risk of discharging patients to a nursing home rather than home. However, the magnitude is relatively small. Data from KOSTRA show that the median of travel distance to the nearest ED in the Northern RHA in 2019 was almost twice as long as that in the South-Eastern health region.^45^ Despite this, the Northern RHA did not exhibit significant differences in admission or discharge patterns. This may be due to confounding between distance and RHA (see Supplemental File 1), where moderate correlations were observed between the 2 variables.

Moreover, our findings show that MIPAC services co-located with GP out-of-hour clinics had an increased relative risk of discharging patients to nursing homes compared to those co-located with nursing homes. Out-of-hour GP clinics offer acute primary healthcare after hours (some 24/7) through both drop-in and telephone consultations, effectively complementing MIPAC services. The lower nursing capacity identified in MIPAC units situated within out-of-hour GP clinics^43^ may partially account for the increased transfer to nursing homes. Given that MIPAC patients are predominantly older adults^8^ and that nursing homes possess specialised expertise in the care of older individuals, MIPAC units located in out-of-hour clinics might lean towards transferring patients to nursing homes, viewing it as a more appropriate option for ensuring continued care. Alternatively, the increased transfer to nursing homes from MIPAC units co-located with GP out-of-hours clinics could also signify the staff’s reduced familiarity with and lower trust in the capabilities of the home care services compared with staff in nursing home-based MIPAC services. We know from previous research that the long-term care services have integrated routines and standards for information exchange and transitional care when patients move between short-term units in nursing homes and the home care services.^46^ Conversely, coordination between the primary care services and the long-term care services is traditionally seen as complicated and inhibited by incompatible electronic patient record systems^47^ and differing institutional logics.^48,49^

Discharge and admission decisions are complex and multifactorial and involve referral criteria, institutional capacity, disease severity and treatment preferences.^10,15^ Although our findings show that MIPAC may be used as hospital step-down care and that over 10% of patients are transferred to the hospital, we do not consider either upward or downward hospital transfers an indication of impaired care quality, efficiency or overall performance of the MIPAC service. Quality assessments should consider patient outcomes and the entirety of patients’ care journeys, rather than being judged based on the direction or frequency of hospital transfers. Patients who are transferred to hospital can benefit from receiving timely MIPAC services in the community setting first. In addition, leveraging MIPAC for step-down care in the case of referrals from the emergency department could serve as an extension of its role in admission avoidance, as well as potentially addressing the challenge of low occupancy in MIPAC units.^50^

Our study suggests considerable facility and regional variations in the admission and discharge rates of patients in the Norwegian MIPAC service. It also highlights the need for further research to understand regional differences in MIPAC care utilisation and triage decisions. However, even though the current study found that 85% of the patients returned to the community after a MIPAC stay, our data did not reveal whether the patients *remained* in the community. Researchers in the United States defined ‘30-day without readmission’ for those discharged to community as ‘successful community discharge’. This was applied as a new quality measure for post-acute inpatient care and further highlighted the need to look beyond the patients’ initial discharge.^13^ Therefore, further studies could extend quality assessments by follow-up of patients to different destinations 30 days after discharge.

To our knowledge, facility and regional variations in MIPAC patients’ admission sources and discharge destinations have not been previously studied. This national-level analysis offers valuable insights for countries with similar admission avoidance intermediate care services based on primary care and for policymakers considering the implementation of new intermediate care models, as it sheds light on how patient trajectories are influenced by various facility and regional characteristics.

## Limitations

Our study had some limitations. First, we did not have data on physician and nursing staff capacity for MIPAC services, which could have been an important supply side variable in our analysis. Previous research has shown that MIPAC units with a 24/7 physician service are associated with a reduction in hospital admissions in the oldest age groups.^33,51^ MIPAC units equipped with 24/7 physician availability may possess greater competence in providing comprehensive diagnosis and treatment, thereby providing patients with appropriate care within the municipality rather than being transferred to a hospital. Hence, the effect of the MIPAC unit’s status with regard to intermunicipal cooperation, MIPAC location, and the number of beds might have been different if we had included a physician availability variable, as the onsite physician could have played an important role in deciding patients’ care trajectories (admission and discharge). However, our study used a random effect to capture unobserved heterogeneity across MIPAC units, which may partially compensate for missing staff information.

The incompleteness of the survey-collected administrative dataset also presents a limitation in the current study. Although a formal power analysis was not conducted prior to the study, the inclusion of 185 out of a population of 215 MIPAC units for the discharge pattern (86% coverage) and 176 out of 215 units for the admission pattern (82% coverage) ensured a comprehensive representation of the population. The high coverage rate and the lack of significant differences between included and missing MIPAC units reduces our concerns about sampling error and enhances the reliability and generalisability of the findings.

## Conclusions

Overall, the Norwegian MIPAC service successfully serves a hospital admission avoidance function. However, it is crucial to highlight the significant variations in its admission and discharge patterns across MIPAC settings and regions, with some MIPAC units receiving and discharging large percentages of their patients from/to hospitals. To explain this variation, our analysis focused on characteristics of the MIPAC service, such as intermunicipal collaboration, the scale and location of MIPAC units, and the health regions in which they were situated. It is evident from our analyses that these factors correlate with where the patients are admitted from and discharged to. This underscores the importance of facility and regional variations as critical elements in assessing healthcare resource allocation and service utilisation, indicating the need for more comprehensive research in this area. Our study suggests that any health service system aiming to implement admission avoidance schemes, such as MIPAC, should consider and close monitor patient transfer trajectories.

## Supplemental Material

sj-docx-1-his-10.1177_11786329241304565 – Supplemental material for Facility and Regional Variations in Admission and Discharge Patterns Within Step-Up Intermediate Care: A Cross-Sectional Study of Municipal Inpatient Acute Care Services in Norway Supplemental material, sj-docx-1-his-10.1177_11786329241304565 for Facility and Regional Variations in Admission and Discharge Patterns Within Step-Up Intermediate Care: A Cross-Sectional Study of Municipal Inpatient Acute Care Services in Norway by Fan Yang, Lisa Victoria Burrell, Maren Kristine Raknes Sogstad and Marianne Sundlisæter Skinner in Health Services Insights

sj-docx-2-his-10.1177_11786329241304565 – Supplemental material for Facility and Regional Variations in Admission and Discharge Patterns Within Step-Up Intermediate Care: A Cross-Sectional Study of Municipal Inpatient Acute Care Services in Norway Supplemental material, sj-docx-2-his-10.1177_11786329241304565 for Facility and Regional Variations in Admission and Discharge Patterns Within Step-Up Intermediate Care: A Cross-Sectional Study of Municipal Inpatient Acute Care Services in Norway by Fan Yang, Lisa Victoria Burrell, Maren Kristine Raknes Sogstad and Marianne Sundlisæter Skinner in Health Services Insights
