# Supplementary material for: Facility and Regional Variations in Admission and Discharge Patterns Within Step-Up Intermediate Care: A Cross-Sectional Study of Municipal Inpatient Acute Care Services in Norway
Source: Health Serv Insights. 2024 Dec 4;17:11786329241304565. doi: 10.1177/11786329241304565 (PMC11618911; doi:10.1177/11786329241304565)
Supplement: sj-docx-1-his-10.1177_11786329241304565 – Supplemental material for Facility and Regional Variations in Admission and Discharge Patterns Within Step-Up Intermediate Care: A Cross-Sectional Study of Municipal Inpatient Acute Care Services in Norway [file sj-docx-1-his-10.1177_11786329241304565.docx]

Supplementary File 1. Correlation across independent variables

|  | Intermunicipal collaboration^*^ | MIPAC unit bed counts^*^ | Location^*^ | RHA^*^ | MIPAC unit's catchment population^*^ | Share of residents aged over 80(%)^**^ | Travel distance between MIPAC host municipality and the nearest emergency department (10km) ^**^ |
| --- | --- | --- | --- | --- | --- | --- | --- |
| Intermunicipal collaboration^*^ | 1 |  |  |  |  |  |  |
| MIPAC unit bed counts^*^ | 0.281 | 1 |  |  |  |  |  |
| Location^*^ | 0.356 | 0.414 | 1 |  |  |  |  |
| RHA^*^ | 0.207 | 0.318 | 0.235 | 1 |  |  |  |
| MIPAC unit's catchment population^*^ | 0.285 | 0.803 | 0.274 | 0.376 | 1 |  |  |
| Share of residents aged over 80(%)^**^ | 0.014 | 0.299 | 0.203 | 0.067 | 0.340 | 1 |  |
| Travel distance between MIPAC host municipality and the nearest emergency department (10km) ^**^ | 0.057 | 0.259 | 0.090 | 0.477 | 0.600 | 0.315 | 1 |

^*^ Cramers’ V test

^**^ Correlation coefficient using linear regression R-square
