# Supplementary material for: Facility and Regional Variations in Admission and Discharge Patterns Within Step-Up Intermediate Care: A Cross-Sectional Study of Municipal Inpatient Acute Care Services in Norway
Source: Health Serv Insights. 2024 Dec 4;17:11786329241304565. doi: 10.1177/11786329241304565 (PMC11618911; doi:10.1177/11786329241304565)
Supplement: sj-docx-2-his-10.1177_11786329241304565 – Supplemental material for Facility and Regional Variations in Admission and Discharge Patterns Within Step-Up Intermediate Care: A Cross-Sectional Study of Municipal Inpatient Acute Care Services in Norway [file sj-docx-2-his-10.1177_11786329241304565.docx]

Supplementary File 2. Pooled Multinomial logistic model

|  | Admission RRR | | | Discharge RRR | | |
| --- | --- | --- | --- | --- | --- | --- |
|  | Nursing home | Hospital | Other municipal facility | Nursing home | Hospital | Other municipal facility |
| Intermunicipal collaboration (ref:independent MIPAC units) | | | | | |  |
| 2-4 municipalities collaborate | **2.11(1.75,2.54)** | **1.36(1.23,1.49)** | **0.10(0.08,0.13)** | 0.99(0.91,1.08) | **1.45(1.33,1.57)** | **0.30(0.24,0.39)** |
| ≥5 municipalities collaborate | 1.11(0.81,1.54) | 0.97(0.87,1.09) | **0.04(0.03,0.06)** | 0.98(0.89,1.07) | **1.45(1.33,1.59)** | **0.59(0.47,0.74)** |
| MIPAC unit bed counts (ref:≤2 beds) | | | |  |  |  |
| 2-5 beds | **0.21(0.17,0.26)** | **1.61(1.38,1.89)** | **8.18(6.17,10.83)** | **0.64(0.58,0.71)** | 1.04(0.94,1.17) | **1.54(1.23,1.93)** |
| >5 beds | **0.12(0.09,0.15)** | **2.06(1.73,2.45)** | **6.88(4.83,9.78)** | **0.49(0.44,0.55)** | 0.96(0.85,1.08) | **0.64(0.48,0.86)** |
| Location (ref: Nursing home) | | |  |  |  |  |
| GP out-of-hours clinic | **0.40(0.30,0.54)** | **1.79(1.55,2.07)** | **0.46(0.35,0.61)** | **0.72(0.65,0.80)** | 1.05(0.94,1.16) | **1.70(1.25,2.32)** |
| Municipal intermediate care institutions | **2.51(2.04,3.1)** | **1.20(1.02,1.40)** | **1.80(1.37,2.38)** | **0.78(0.70,0.87)** | **0.77(0.69,0.86)** | **4.06(3.14,5.25)** |
| Other facility | 0.95(0.69,1.31) | **6.76(5.82,7.84)** | **14.15(10.37,19.29)** | 0.91(0.81,1.02) | 0.98(0.86,1.10) | **4.69(3.45,6.38)** |
| Multiple locations | **1.87(1.52,2.32)** | 0.89(0.76,1.03) | **0.37(0.30,0.47)** | **0.55(0.50,0.61)** | 1.07(0.97,1.19) | **1.47(1.15,1.88)** |
| RHA (ref: South-Eastern Norway RHA) | | | |  |  |  |
| Western Norway RHA | 0.97(0.79,1.20) | **0.58(0.53,0.64)** | **0.02(0.01,0.03)** | **0.82(0.76,0.89)** | **1.24(1.14,1.34)** | **0.27(0.20,0.37)** |
| Middle Norway RHA | 1.15(0.94,1.41) | **0.68(0.58,0.79)** | **0.09(0.04,0.18)** | **1.24(1.13,1.37)** | **0.83(0.74,0.93)** | **1.37(1.08,1.73)** |
| Northern Norway RHA | **2.60(2.15,3.14)** | **0.77(0.67,0.89)** | **0.31(0.23,0.41)** | **0.86(0.77,0.95)** | 1.05(0.94,1.18) | **0.60(0.47,0.76)** |
| Share of residents aged over 80 | 1.01(0.94,1.08) | 1.03(0.98,1.08) | 1.04(0.92,1.17) | 0.97(0.93,1.00) | 0.97(0.93,1.01) | **1.39(1.27,1.51)** |
| Travel distance between MIPAC host municipality and the nearest emergency department (10km) | **0.94(0.92,0.95)** | **1.04(1.03,1.05)** | **1.08(1.07,1.10)** | **0.98(0.97,0.98)** | **1.04(1.03,1.05)** | **1.06(1.04,1.07)** |
| Constant | **0.07(0.05,0.11)** | **0.04(0.03,0.05)** | **0.01(0.01,0.03)** | **0.66(0.53,0.81)** | **0.18(0.15,0.23)** | **0.01(0,0.01)** |

*P<0.05 is in boldface
